# Anesthetic management in pregnant women with Fontan circulation: a case series

**DOI:** 10.1186/s40981-024-00706-3

**Published:** 2024-04-18

**Authors:** Ai Fujita, Kazuhiro Shirozu, Midoriko Higashi, Ken Yamaura

**Affiliations:** 1 Operating Rooms, Kyushu University Hospital, Fukuoka, Japan; 2 Department of Anesthesiology and Critical Care Medicine, Graduate School of Medical Sciences, Kyushu University, Fukuoka, Japan; 3 Department of Anesthesiology, Fukuoka Children’s Hospital, Kashiiteriha 5-1-1, Higashi-ku, Fukuoka, 813-0017 Japan

**Keywords:** Fontan circulation, Mode of delivery, Anesthetic management, Labor analgesia, Anticoagulation therapy

## Abstract

**Background:**

Given the advances in medicine, women with Fontan circulation are now reaching childbearing age. However, data on the mode of delivery and anesthetic management of these patients are limited. We report the cases of five pregnant women with Fontan circulation.

**Case presentation:**

The mean age at delivery was 28 ± 3 years, and the mean gestational period was 34 weeks and 3 days. Anticoagulation therapy was switched from warfarin and aspirin to continuous intravenous heparin. The modes of delivery were scheduled cesarean section (C/S) in one, emergency C/S in three, and vaginal delivery with epidural labor analgesia in one patient. Three patients underwent C/S under regional anesthesia; one received general anesthesia. The perinatal complications were heart failure, worsening valve regurgitation, and postoperative hematoma in three, four, and two patients, respectively.

**Conclusions:**

For C/S in women with Fontan circulation, regional anesthesia should be considered. Epidural labor analgesia can help prevent the decrease in pulmonary blood flow due to straining. We initiated labor analgesia or C/S with regional anesthesia at the appropriate time in four patients.

## Background

Owing to advances in surgical and medical management, women with Fontan circulation are reaching childbearing age. Fontan physiology is characterized by a single ventricle, with blood flow into the pulmonary arteries occurring passively without the assistance of an intervening pump [1]. These patients are at an increased risk of maternal morbidities during labor and delivery, including heart failure, arrhythmia, hypertensive disorders of pregnancy, and postpartum hemorrhage [2, 3]. Such cases have been reported previously [4–6]; however, perioperative management including anesthesia and analgesia methods and postoperative anticoagulation therapy has not been standardized. We report the cases of pregnancy and delivery in five women with Fontan circulation.

## Case presentation

### Baseline characteristics

The primary diseases were pulmonary artery obstruction in one patient and tricuspid valve obstruction in four patients. Between 1991 and 2001, three and two patients underwent the Fontan procedure with a lateral tunnel (LT) and with an extracardiac connection (EC), respectively. The baseline oxygen saturation (SpO_2_) was slightly low in two patients (patients 3 and 5). None of the patients experienced Fontan failure. Four patients had mild valve regurgitation, and patient 3 had a small amount of LT leakage (Table 1).

**Table 1:** Baseline characteristics of the five patients

Case	Age (years)	Initial diagnosis	Fontan operation type	NYHA class	SpO_2_ (%)	RpI (wood unit/m^2^)	CVP (mmHg)	EF (%)	Cardiac complications	Infertility treatment
1	24	PA, AVSD, TAPVC	EC	I	97	1.06	10	57	Mild AVVR	Ovulation induction
2	30	TA	LT	I	96	N/A	N/A	56	Mild AVVR	–
3	29	TA	LT	I	92	0.67	8	54	Mild LT leakage	–
4	32	TA	LT	II	97	1.23	9	54	Mild MR	Twin pregnancy after AIH
5	25	TA	EC	I	90	1.39	11	64	Trivial MR	–

*NYHA* New York Heart Association, *SpO*_*2*_ Oxygen saturation, *RpI* Pulmonary vascular resistance index, *CVP* Central venous pressure, *EF* Ejection fraction, *PA* Pulmonary atresia, *AVSD* Atrioventricular septal defect, *TAPVC* Total anomalous pulmonary venous connection, *EC* Extracardiac connection, *AVVR* Atrioventricular valve regurgitation, *TA* Tricuspid atresia, *LT* Lateral tunnel, *N/A* Not available, *MR* Mitral regurgitation, *AIH* Artificial insemination of husband

### Anticoagulation therapy

Once pregnancy was detected, all patients discontinued warfarin and switched to 10,000 U/day of subcutaneous calcium heparin. Aspirin 100 mg/day was continued until the start of the intravenous heparin injection. While patient 3 was admitted at 35 weeks of gestation, the rest were admitted at 22–25 weeks of gestation; subcutaneous calcium heparin was switched to intravenous unfractionated heparin starting dose of 10,000–15,000 U/day adjusted to APTT of 45–80 s (10,000–35,000 U/day). Intravenous heparin was administered until just before delivery. After confirming the absence of bleeding during delivery, intravenous heparin was resumed. Warfarin was resumed on 1–3 days postpartum. Intravenous heparin was switched to aspirin at 4–6 days postpartum.

### Mode of delivery and anesthetic management

The mean age at delivery was 28 ± 3 years. All patients were primiparas with a mean gestational period of 34 weeks and 3 days at delivery. All patients delivered preterm infants. The modes of delivery were scheduled cesarean section (C/S) in one, emergency C/S in three, and vaginal delivery with epidural labor analgesia in one patient. Three patients underwent C/S under regional anesthesia while one received general anesthesia.

#### Patient 1

She was provided epidural labor anesthesia with an initial dose of 10 mL of 0.125% levobupivacaine and 50 μg of fentanyl, followed by continuous epidural infusion of 10 mL/h of 0.125% levobupivacaine including 1 μg/mL of fentanyl. The patient’s hemodynamics remained stable throughout the intrapartum period.

#### Patient 2

This patient was the first woman with Fontan circulation to deliver a newborn at our hospital. She was scheduled for C/S under combined spinal and epidural anesthesia with arterial blood pressure and central venous pressure (CVP) monitoring in addition to standard monitoring. Fluid management was performed to maintain a CVP of approximately 10 mmHg during the operation. Postoperatively, the patient was managed in the intensive care unit. On the day of delivery, her urine output decreased and CVP increased to 14 mmHg but improved after diuretic administration.

#### Patient 3

The patient developed heart failure symptoms including exertional dyspnea and tiredness at 34 weeks of gestation and required oxygen. She underwent emergency C/S under combined spinal and epidural anesthesia at 35 weeks due to worsening heart failure symptoms. After delivery, continuous diuresis was achieved without the use of diuretics, and the heart failure symptoms improved. Home oxygen therapy (HOT) was required for exertional oxygen desaturation.

#### Patient 4

The patient developed heart failure symptoms at 26 weeks of gestation and required oxygen, diuretic administration, and red blood cell transfusion. She had massive genital bleeding at 29 weeks of gestation due to pregnancy with twins and placenta previa, resulting in emergency C/S under spinal anesthesia. Her heart failure symptoms persisted post-partum. These symptoms gradually improved with diuretics and the resumption of carvedilol. HOT was required because of oxygen desaturation.

#### Patient 5

The patient went into labor at 34 weeks and underwent emergency C/S because of the pelvic position of the fetus. After neutralization with protamine, the patient underwent general anesthesia because activated partial thromboplastin time (APTT) results were not available. Rapid sequence induction was performed with propofol 110 mg and rocuronium 50 mg. We adjusted pressure control ventilation (PCV) to set the peak pressure at 14 cmH_2_O and positive end-expiratory pressure at 4 cmH_2_O. After induction of anesthesia, her blood pressure decreased but improved after the administration of phenylephrine, calcium gluconate, and infusion loading with colloidal fluid.

In patients 2–4, spinal anesthesia was performed with 2.0–2.5 mL of 0.5% isobaric bupivacaine. Their hemodynamics were stable during the intraoperative period without continuous administration of inotropic agents (Table 2).

**Table 2:** Summary of deliveries and anesthetic management

Case	Gestation at delivery (weeks)	Mode of delivery	Labor analgesia was scheduled	Indication for C/S	Neutralization by protamine	Anesthesia management	Monitoring	Fluids	Blood loss (mL)	Intake/output balance (mL)	Use of vasopressor
Vaginal delivery											
1	34	Labor analgesia	+		+	Epi	Standard	N/A	500	N/A	No
C/S											
2	36	Scheduled C/S	–		-	Epi, Spi	Standard, A, CVP	800 mL crystalloid, 1500 mL colloid	1120	Positive 860	No
3	35	Emergency C/S	+	Worse CHF	-	Epi, Spi	Standard	1200 mL crystalloid	209	Positive 793	No
4	29	Emergency C/S	–	Twin pregnancy, placenta previa	–	Spi	Standard, A	700 mL crystalloid	1482	Negative 952	Phenylephrine 0.1 mg
5	34	Emergency C/S	+	Breech presentation	+	General anesthesia	Standard	860 mL crystalloid, 400 mL colloid, 140 mL RBC	1410	Negative 209	Phenylephrine 0.4 mg, calcium gluconate 15 mL

*C/S* Cesarean section, *Epi* Epidural anesthesia, *N/A* Not available, *Spi* Spinal anesthesia, *A* Arterial blood pressure, *CVP* Central venous pressure, *CHF* Cardiac heart failure, *RBC* Red cell concentrate

### Postpartum complications

Four (80%) patients experienced exacerbations of the New York Heart Association (NYHA) functional class. Three patients (60%) had heart failure symptoms requiring oxygenation, while two patients (40%) required HOT. Four patients (80%) experienced worsening valve regurgitation. Two patients (40%) experienced postoperative bleeding and had to be temporarily withdrawn from anticoagulation therapy. In patient 2, genital bleeding occurred on the fifth postpartum day using intravenous heparin, aspirin, and warfarin. In patient 4, a lower abdominal hematoma was found on the fifth postpartum day using intravenous heparin and warfarin (Table 3). None of the patients developed arrhythmia during the gestational or postpartum periods.

**Table 3:** Summary of postpartum periods

Case	NYHA class	SpO_2_ (%)	EF (%)	Complications	Heart failure symptoms	Use of diuretic
Vaginal delivery						
1	I	96	N/A	Worse AVVR, liver congestion	–	No
C/S						
2	II	95	68	Moderate MR, genital bleeding	–	Furosemide, spironolactone
3	IV	90 (O_2_ 1L)	60	Requiring HOT, liver congestion	+	No
4	III	97 (O_2_ 1L)	47	Worse MR, requiring HOT, hematoma	+	Furosemide
5	II	93 (O_2_ 1L)	N/A	Worse MR	+	Furosemide

*C/S* Cesarean section, *NYHA* New York Heart Association, *SpO*_*2*_ Oxygen saturation, *EF* Ejection fraction, *AVVR* Atrioventricular valve regurgitation, *MR* Mitral regurgitation, *HOT* Home oxygen therapy

## Discussion

Although several perinatal outcomes of pregnancies in patients with Fontan circulation have been reported [3–6], there is no consistent view on the mode of delivery or anesthetic management. Herein, we summarized the clinical data of five pregnant women with Fontan circulation, with a discussion of the literature.

Blood loss, blood volume shift, and thrombotic complications have been reported to increase with C/S [7, 8]. Ruys et al. recommended planned vaginal delivery because of the lack of advantages or adverse fetal outcome with planned cesarean section compared with planned vaginal delivery in patients with congenital heart diseases [9]. However, approximately half of the patients with Fontan circulation undergo C/S, usually for obstetric indications [8]. In the present series, four patients (80%) were scheduled for C/S. Patient 2 was planned for C/S; however, vaginal delivery may have been possible. Epidural labor analgesia was planned for three patients, but only one painless delivery was achieved. C/S is often selected in cases of severe heart failure. If the condition of the fetus permits, labor analgesia may be planned before the deterioration of heart failure symptoms.

Three patients who underwent C/S received spinal subarachnoid anesthesia. Even with regional anesthesia, preload reduction due to sympathetic blockade may occur, resulting in decreased pulmonary blood flow and cardiac output (CO). To prevent rapid preload reduction, appropriate infusion loading and small divided doses with epidural anesthesia are recommended [4, 8]. In our patients, spinal subarachnoid anesthesia with the minimal use of local anesthetics resulted in stable intraoperative hemodynamics. With the use of general anesthesia, the blood pressure temporarily dropped after induction. General anesthesia can cause a decrease in systemic vascular resistance (SVR), and PCV causes a decrease in venous return and increase in pulmonary vascular resistance (PVR). Maintaining preload is essential to managing Fontan circulation. It is generally known that spinal anesthesia causes strong vasodilation and greater preload reduction than general anesthesia. In this case series, however, the patient under general anesthesia became hypotensive, which was difficult to manage. It was thought to be due to the large amount of anesthetics. Adequate fluid loading and continuous administration of inotropic agents before anesthesia induction may have prevented the sudden decrease in SVR. Regional anesthesia is preferred to avoid these risks [5, 6]. Phenylephrine, ephedrine, and norepinephrine are commonly used as vasoconstrictor and inotropic agents [4–6]. Phenylephrine can also increase the PVR and should be used with caution in Fontan-palliated patients [5]. However, invasive monitoring is not always necessary. The number of reports of good outcomes without the use of invasive monitoring has increased in recent years [4, 5]. Central venous access is not encouraged owing to the risk of thromboembolic complications [6].

After delivery, the intravascular volume increases due to uterine contractions, posing the risks of heart failure and arrhythmia. The hemodynamics and intake–output balance should be evaluated for appropriate fluid management. In patient 4, the heart failure symptoms persisted postpartum. The CO has been reported to be higher in twin pregnancies than in singleton pregnancies [10], and Fontan-palliated patients have a limited ability to increase CO and adapt to these changes.

Anticoagulation management in patients with Fontan circulation is important because these patients are prone to thrombosis owing to increased coagulability caused by specific venous stasis [11]. Adjustment of anticoagulants during pregnancy is difficult, particularly when administering regional anesthesia. In previous reports, antiplatelet therapy with aspirin or anticoagulation with heparin was used in 45–100% of the patients [3–6]. Intravenous heparin is highly adjustable owing to its short half-life and neutralization by protamine. At our hospital, intravenous heparin is administered as inpatient management, starting from the second trimester of pregnancy, discontinued before delivery, and neutralized with protamine when necessary. Anticoagulation therapy was resumed postpartum but was temporarily interrupted because of bleeding in two patients. The APTT and prothrombin time-international normalized ratio were regularly monitored; however, the patients were considered to be in a state of temporary bleeding. In both patients, postpartum hemorrhage was stopped by temporary withdrawal of anticoagulation therapy. In patient 3, intravenous heparin was discontinued when warfarin was initiated. Therefore, the anticoagulants should be switched with caution.

Care should be provided to pregnant women with Fontan circulation by an interdisciplinary team of obstetricians, cardiologists, pediatricians, and anesthesiologists for determining the timing and method of delivery and anesthesia. Regional anesthesia during labor or C/S should be initiated at the appropriate time through heparin discontinuation, neutralization with protamine, and rapid APTT measurement. In the postpartum period, patients should be monitored for cardiac failure, arrhythmia, hypoxia, and postpartum hemorrhage.

## Data Availability

Data relevant to this case series are not available for public access because of patient privacy concerns but are available from the corresponding author upon reasonable request. Ethics approval and consent to participate Not applicable. Consent for publication Written informed consent was obtained from the patient for the publication of this case series and the accompanying images.

## Abbreviations

APTT: Activated partial thromboplastin time
CO: Cardiac output
C/S: Cesarean section
CVP: Central venous pressure
EC: Extracardiac connection
HOT: Home oxygen therapy
LT: Lateral tunnel
PVR: Pulmonary vascular resistance
SVR: Systemic vascular resistance
